# Sex differences in entrapment in a multinational sample: a network analysis perspective

**DOI:** 10.3389/fpsyt.2024.1321207

**Published:** 2024-05-28

**Authors:** Cristian Ramos-Vera, Dennis Calle, Gleni Quispe-Callo, Inken Höller, Thomas Forkmann, Jorge Ordoñez-Carrasco, Radka Čopková, Vladimir Lichner, Marlon Lobos-Rivera, Yaquelin E. Calizaya-Milla, Jacksaint Saintila

**Affiliations:** ^1^ Área de Investigación, Universidad César Vallejoo, Lima, Peru; ^2^ Escuela de Psicología, Universidad Nacional de San Agustín de Arequipa, Arequipa, Peru; ^3^ Department of Clinical Psychology and Psychotherapy, University of Duisburg-Essen, Essen, Germany; ^4^ Department of Clinical Psychology and Psychotherapy, Charlotte Fresenius Hochschule, Düsseldorf, Germany; ^5^ Departamento, Psicología, Universidad de Almería, Almería, Spain; ^6^ Faculty of Economics, Technical University of Košice, Košice, Slovakia; ^7^ Department of Social Work, Faculty of Arts, Pavol Jozef Šafárik University in Košice, Košice, Slovakia; ^8^ Escuela de Psicología, Universidad Tecnológica de El Salvador, San Salvador, El Salvador; ^9^ Facultad de Ciencias de la Salud, Universidad Peruana Unión, Lima, Peru; ^10^ Facultad de Ciencias de la Salud, Universidad Señor de Sipán, Chiclayo, Peru

**Keywords:** entrapment, network analysis, internal entrapment, external entrapment, gender differences, cross-cultural differences

## Abstract

**Background:**

The concept of entrapment has been highlighted as a transdiagnostic element that manifests itself in disorders such as depression, anxiety, and suicidal ideation. Although research has been conducted in different contexts independently, a comprehensive multi-country study to assess gender differences in entrapment through network analysis has not yet been carried out. The objective of this study was to evaluate the entrapment network in men and women at the multinational level.

**Methods:**

A sample of 2,949 participants, ranging in age from 18 to 73 years from six countries (Germany, Iran, Spain, Slovakia, El Salvador, and Peru), was considered. They completed the entrapment scale. A network analysis was performed for both men and women to identify the connectivity between indicators and the formation of clusters and domains, in addition to the centrality assessment in both sex groups.

**Results:**

The study findings revealed the presence of a third domain focused on external interpersonal entrapment in the network of men and women. However, in relation to the interconnectivity between domains, variations were evidenced in both networks, as well as in centrality, it was reported that men present a greater generalized entrapment in various aspects of life, while women tend to experience a more focused entrapment in expressions of intense emotional charge.

**Conclusion:**

The multinational study identified variations in the structure of entrapment between genders, with three domains (internal, external, and external-interpersonal) and differences in the interaction of indicators and groupings, as well as discrepancies in centrality.

## Introduction

Entrapment describes the feeling of being trapped in an unpleasant or stressful state or situation with no possibility of escape (1). The concept of entrapment has been linked to a variety of mental disorders, among which anxiety and post-traumatic stress disorders (PTSD; 2) stand out. However, its relevance lies in its strong connection with depressive disorders and suicide-related behaviors (1, 3). Consequently, entrapment has been integrated as a central construct in several theories that strive to explain the development of depression and suicidal ideation. It is part of the Cry-of-Pain-Model (4) and the main pathway of the Integrated Motivational-Volitional Model of Suicidal Behavior (5). In general, entrapment has been discussed as a transdiagnostic construct (2).

Gilbert & Allan (1) further propose that entrapment can be differentiated into two sub-aspects, namely, internal and external entrapment. Internal entrapment refers to the feeling of being trapped by internal factors such as who one perceives themselves to be. This phenomenon has been emphasized as a predictor of suicidal ideation in hospitalized individuals with an increased susceptibility to suicide (6, 7). External entrapment, on the other hand, is defined as being trapped due to external circumstances such as relationship problems or job loss (3). These two dimensions have been conceptually validated in the study by Gilbert & Allan (1), and the same results were replicated in three more recent studies in the United Kingdom (8) and two studies in Germany, with normative (9) and clinical samples (7).

To date, there are few studies that evaluate the existence of significant sex differences in levels of feelings of entrapment suggesting that women exhibit higher levels of entrapment compared to men. Examples of such research include the study by Choi & Shin (10), which examined 367 participants in South Korea, and the study by Ren et al. (11), which involved 1,074 participants in China, both studies highlight that according to psychosocial factors, such as abusive relationships, unequal opportunities, gender roles, it is women who demonstrate greater entrapment compared to men. On the other hand, Siddaway et al. (2) conducted a systematic review and meta-analysis in which they analyzed 40 publications involving 10,072 participants. They found that the feeling of entrapment acts as a moderator in several psychiatric disorders. Furthermore, in 34 of these studies, involving participants from Europe and Asia, it was observed that women reported higher levels of entrapment. However, the scientific literature shows contradictory results, as there is research that argues that there are no notable sex-related differences in feeling of entrapment. For example, research conducted in the UK context, such as Griffiths’ (12) study involving 169 participants, suggests that the perception of entrapment is equivalent between men and women. Gilbert & Allan (1) reported another similar finding with a sample of 392 individuals, of whom 302 were university students and 90 were clinical patients.

One of the main risk factors for suicidal ideation is perceived burdensomeness, which is the belief that one is a burden to others, that one’s existence causes more problems than benefits to those around them (13). Women are likely to be more likely to internalise social conflicts and expectations, which can lead to greater self-blame and ultimately a perception of being a burden to others. Furthermore, ideals of femininity that emphasize altruism and concern for others may increase the pressure on women to meet the needs of others, which can also contribute to feelings of burden. Women may also be more exposed to these thinking patterns due to social and cultural pressures that emphasize the importance of fulfilling multiple roles, such as mother, wife, sister, daughter, professional, and family caregiver (14). Men are expected to be strong, independent, and emotionally resilient, which can make it difficult for them to express feelings of vulnerability or seek help when they are struggling. This may contribute to a greater sense of strain, as they may feel that they are not living up to social and family expectations, in addition to the fact that, unlike women, they tend not to have a reliable social support network (15).

To date, no studies have been documented that have examined sex differences between external and internal entrapment; the relevance of reporting these differences lies in the need for a comprehensive understanding of these dimensions that make up entrapment. This provides a more detailed understanding of how this factor should be interpreted from the perspectives of men and women. In addition, it is important to determine whether one or both dimensions of entrapment occur unequally between the sexes. This is essential to inform and guide policy and decision making, allowing the design of more specific prevention and promotion programs tailored to the particularities and requirements of each gender.

A significant advance in various disciplines of psychology is the adoption of the network approach (16, 17). This multivariate model represents psychological phenomena as a network system, where psychological characteristics emerge from the reciprocal interconnections between components or indicators of a psychological variable (e.g., items or subscales) (18). Network models provide an alternative perspective to assess psychological measures without the need to identify a common latent variable.

This approach uses a Gaussian graph to represent the associations (edges; total or partial correlations) existing between the variables (nodes; 19, 20). Furthermore, it includes centrality indices that quantify the topological characteristics of components within the network (21). In the network model, entrapment is denoted as a system that emerges due to the interaction between the indicators that together form the construct. These indicators are not simply a cause or by-product of entrapment, as depicted in factor models. Instead, direct interactions between item responses form the systemic structure of the construct. Specifically, entrapment may not cause the appearance of its internal and external facets. Rather, entrapment emerges because of interactions between these factors or their respective items according to network models (19, 22, 23).

This approach provides additional information on the explanatory characteristics of the causal structure represented by the activation of the relationships between the elements of the psychological phenomenon of entrapment that requires its evaluation from another quantitative model to explore new findings at the multinational level (24, 25). For example, depending on the topological structure of the network with different degrees of connection and position of the nodes, it is possible to determine the grouping of the items and their possible overlapping to more than one domain (26–28). Other more essential quantitative results are shared centrality and shared variance, denoting a more important role of such indicators in the systemic manifestation of the network.

To date, there is a lack of research that includes samples from Latin American contexts, especially research that applies the methodology of psychological networks. At the same time, previous studies conducted in other contexts were not evaluated in terms of relevant sociodemographic factors, such as gender, and previous studies presented samples limited to one cultural context and language. Therefore, the aim of this study was to perform the representation of network models as a function of sex in a larger and more diverse multi-national sample to gain a better understanding of the interaction and clustering of indicators from the perspective of these multivariate systemic models that allow the representation of psychological entrapment.

## Materials and methods

### Sample

The sample consisted of participants from six countries, including El Salvador (852; 53.74% female and 46.26% male), Germany (454; 75.6% female and 24.4% male), Iran (306; 66.7% female and 33.3% male), Spain (635; 51.8% female and 47.9% male), Slovakia (393; 67.4% female and 32.6% male) and Peru (309; 44% female and 56% male). Regarding the description of sociodemographic aspects, 53.24% of the participants were men (n=1,570) and 46.8% were women (n=1,379) 2,949. Similarly, the samples were made up of 46% adult university students (n=1,356) and 54% nonuniversity students (n = 1,593), with respect to partners, 18% reported having a current partner (n=521), 19.3% reported not having a partner (n=568), while 63.1% reported no information on marital status (n=1860), 10.2% were employed (n=301), 11.3% of the participants did not work (n=334) and 78.5% did not answer this information (n=2,314). Finally, for the collection of the sample, convenience sampling was considered, considering as eligibility criteria participants who were 18 years of age or older, a minimum educational level of secondary school or high school, and that the evaluation would be carried out during the period 2021 to 2023.

### Instrument

Entrapment Scale (ES): The Entrapment Scale (ES; 1) is composed of 16 items that assess the perception of being trapped in intense, highly stressful internal circumstances with no perceived opportunity for escape. An example item on the scale is “I would like to escape from my own thoughts and feelings.” Measurement is a psychometric instrument based on self-report. Participants were asked to rate their emotional state during the past week using a five-point Likert scale, ranging from 0 (“not at all like me”), indicating no presence of entrapment, to 4 (“extremely like me”), indicating high presence of entrapment. The ES was validated in Germany, Spain, and Iran (29–31). The translation of the ES was performed in the other three countries (that is, Slovakia, El Salvador, and Peru). The initial translation was reviewed in collaboration with two other researchers: disagreements and differences were discussed as a group to refine the scale in both applied languages (i.e., Spanish and English) for application to the Latin American and Slovak context. The process required cross-linguistic equivalence based on back-translation (32, 33), to know the suitability with the original scale, and to have the versions used in the present study for the data survey.

### Procedures

The study team used a standardized form, composed of relevant research information, informed consent, entrapment scale, which was adapted to be used in virtual platforms of social networks (Facebook, Whatsapp) and online survey platforms such as Qualtrics, Google Forms, and SosciSurvey. Data were collected in 2021 in Germany, Spain, Iran, and Peru, while in Slovakia and El Salvador it was in 2023, respectively. Regarding language adaptation, countries whose participants were surveyed in a language other than English had the Entrapment Scale translated into their respective languages (Persian, German, and Spanish) by a translator and clinical psychology researcher, who certified the appropriate translation and adaptation to the context.

### Statistical analyses

The network models were based on partial correlations in both samples and estimated with the ‘huge’ package (34), which allows for a nonparanormal transformation of the data in the RStudio software. In networks, nodes represent variables, and edges (lines) signify partial correlations between these variables. These partial correlations enable multivariate control while accounting for relationships with all other nodes, helping to avoid spurious correlations. Furthermore, the absence of an edge between nodes indicates that the two variables are conditionally independent. All these connections were visualized with the qgraph package (35). To evaluate the precision of correlation networks, we performed 2000 nonparametric bootstrapping iterations using the bootnet package (36). Furthermore, we computed centrality values in both networks, including expected influence (relevant connections within the global network), bridge expected influence (important connections between communities), and predictability (nodes that are strongly predicted by neighboring nodes). The purpose of this analysis was to identify entrapment items that play a crucial role in forming strong connections throughout the network, with the goal of recognizing and mitigating their impact in future interventions (37, 38). In this research, we also sought to identify trapping items that belong to several overlapping communities. To achieve this, we employed the Clique Percolation Method. (39). This method is valuable because it allows nodes to belong to multiple groups based on the content of items. Identifies fully connected groups of neighboring nodes, known as k-cliques. By using a permutation test, we established two essential parameters for clique percolation: “k” determines clique size and “I” measures clique connectivity strength for community identification. We tested various configurations by setting “k” values between 3 and 8 and allowing “I” values up to 0.09, providing a range of node groupings to explore. Finally, the Network Comparison Test (NCT; 40) was applied to assess whether significant differences exist in network structure, global network strengths, and edges across sex-based networks. The results of this method were tested using 1000 iterations.

## Results

**Table 1** shows the descriptive statistics for men and women. On the one hand, in relation to men where item 6 shows higher scores (“I feel trapped by my obligations”) and item 13 (“I would like to escape from my thoughts and feelings”) with a mean of 1.50 (SD = 1.35) and 1.40 (SD = 1.41) respectively. On the other hand, in women the highest scoring items are item 5 (“I feel powerless to change things”) and item 6 (“I feel trapped by my obligations”) with a mean of 1.41 (SD =1.32) and 1.30 (SD = 1.30). Regarding skewness and kurtosis, the data evidence in both sexes a univariate nonnormality since their values exceed the interval of +/- 1.5 (41). The items with the highest predictability values (r2) in both groups were items 14 (men: r2 = 0.74; women: r2 = 0.79), 16 (men: r2 = 0.73; women: r2 = 0.75) and 11 (men: r2 = 0.72; women: r2 = 0.75).

**Table 1:** Descriptive statistics of the sample according to sex.

Item	Male (n=1,570)					Female (n=1,379)				
	M	SD	Skewness	Kurtosis	r^2^	M	SD	Skewness	Kurtosis	r^2^
1	1.16	1.30	0.81	-0.57	0.63	1.18	1.31	0.76	-0.68	0.68
2	1.20	1.35	0.75	-0.77	0.67	1.06	1.27	0.92	-0.35	0.71
3	0.77	1.18	1.57	0.82	0.55	0.99	1.25	1.01	-0.19	0.54
4	1.24	1.40	0.73	-0.86	0.71	1.24	1.35	0.71	-0.80	0.73
5	1.39	1.35	0.54	-0.99	0.63	1.30	1.30	0.63	-0.79	0.67
6	1.50	1.35	0.43	-1.05	0.54	1.41	1.32	0.52	-0.91	0.55
7	1.11	1.31	0.86	-0.52	0.71	1.13	1.28	0.82	-0.54	0.69
8	0.91	1.23	1.17	0.22	0.56	1.15	1.31	0.80	-0.61	0.53
9	1.22	1.40	0.78	-0.77	0.64	1.23	1.40	0.75	-0.80	0.72
10	0.86	1.20	1.23	0.40	0.63	0.99	1.25	0.99	-0.26	0.64
11	1.14	1.37	0.86	-0.62	0.72	1.08	1.32	0.91	-0.50	0.75
12	1.26	1.32	0.66	-0.82	0.56	1.28	1.31	0.63	-0.83	0.64
13	1.40	1.41	0.55	-1.05	0.68	1.33	1.36	0.64	-0.87	0.71
14	1.12	1.36	0.88	-0.58	0.74	1.07	1.32	0.94	-0.41	0.79
15	1.20	1.42	0.81	-0.76	0.68	1.29	1.42	0.68	-0.91	0.71
16	0.99	1.33	1.07	-0.21	0.73	1.02	1.31	0.99	-0.33	0.75
Omega reliability by country										
Peru									0.98	
Germany									0.96	
El Salvador									0.96	
Spain									0.96	
Slovakia									0.95	
Iran									0.94	

r^2^: predictability of networks.

**Figure 1:**
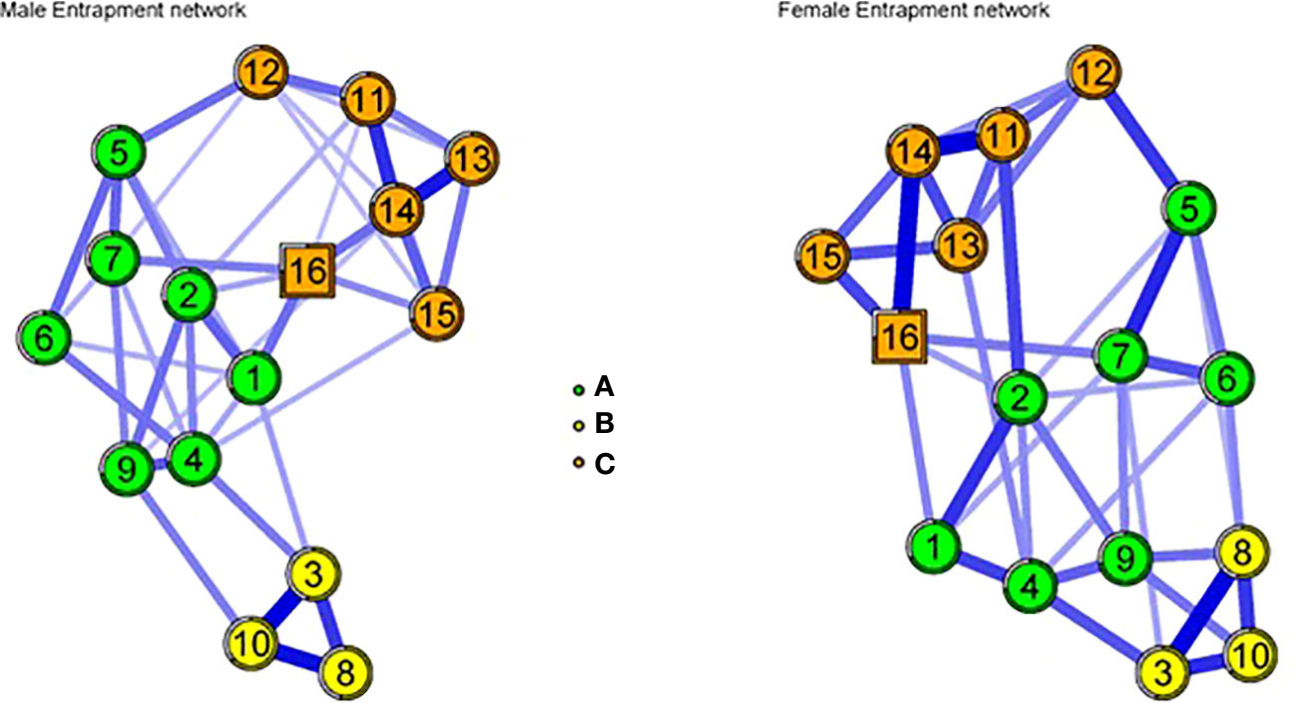
Network analysis in men and women. A= group 1 (7 external entrapment items); B= group 2 (3 external - interpersonal entrapment items). C= group 3 (6 internal entrapment items). Square-shaped nodes in both networks represent the highest centrality (bridge-expected influence). Item1: I am in a situation I feel trapped in, item 2: I have a strong desire to escape from things in my life, item 3: I am in a relationship I cannot get out of, item 4: I often have the feeling that I would just like to run away, item 5: I feel powerless to change things, item 6: I feel trapped by my obligations, item 7: I can see no way out of my current situation, item 8: I would like to get away from other more powerful people in my life, item 9: I have a strong desire to get away and stay away from where I am now, item 10: I feel trapped by other people, item 11: I want to get away from myself IE, item 12: I feel powerless to change myself, item 13: I would like to escape from my thoughts and feelings, item 14: I feel trapped inside myself IE, item 15: I would like to get away from who I am and start again, item 16: I feel I’m in a deep hole I cannot get out of.

However, in **Figure 1** the items of the male and female networks were observed to be grouped into three domains that refer to external entrapment (A), internal entrapment (B), and external-interpersonal entrapment (C). The strongest relationships in the men’s network were between indicators of external-interpersonal entrapment indicators such as item 3 “I am in a relationship I cannot get out of” and item 10 “I feel trapped by other people” (r = 0.30, p < 0.01), as well as between items 8 “I would like to get away from other more powerful people in my life” and item 10 “I feel trapped by other people” (r = 0.28, p < 0.01) which belong to the same group mentioned. Regarding the female network, stronger associations were observed between external entrapment indicators such as item 11 “I want to escape from myself IE” and item 14 “I feel trapped inside myself IE” (r = 0.25, p<0.01), also between external-interpersonal entrapment indicators such as item 3 “I am in a relationship I cannot escape” and 8 “I would like to escape from other more powerful people in my life”. All remaining correlations for both networks are available in the **Supplementary Material (Tables A, B)**.

**Figure 2:**
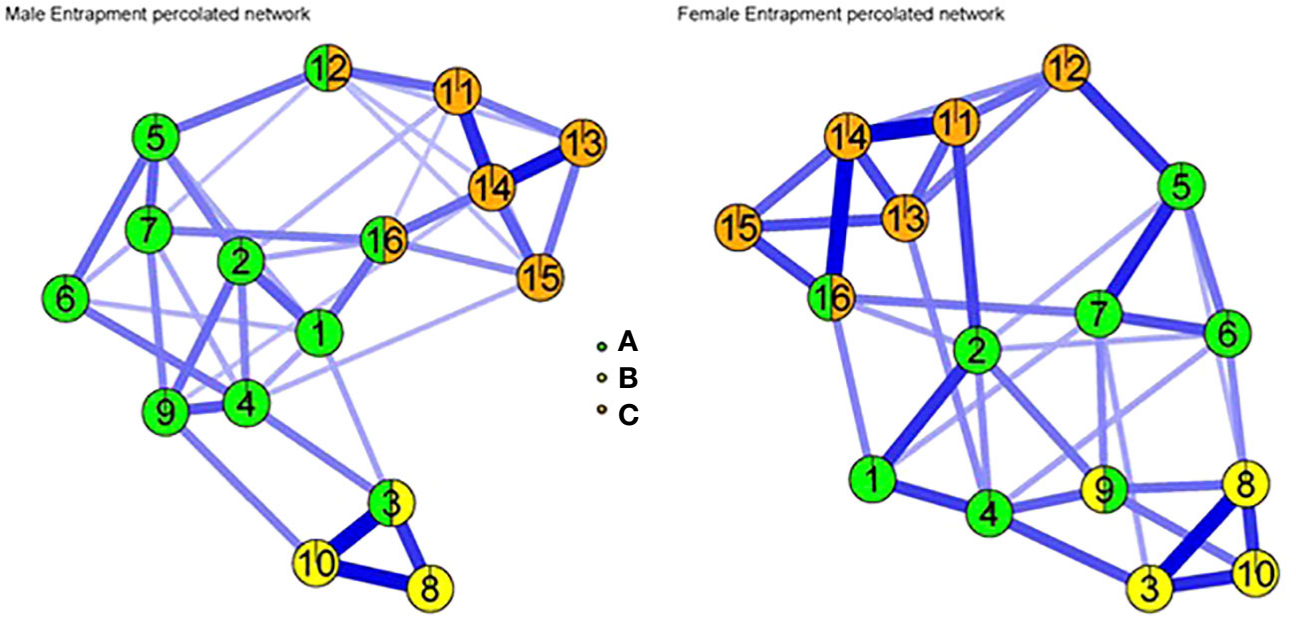
Network analysis with percolation in men and women. In the male network, Items 12 and 16 belong to two clusters simultaneously of external entrapment (7-item cluster) as well as internal entrapment (6-item cluster) items, while item 3 overlapped both facets of external entrapment and external-interpersonal entrapment. In the female network, item 16 was part of the external and external-interpersonal (7-item and 3-item cluster) and internal clusters simultaneously, while item 9 overlapped with the two domains of external and external-interpersonal entrapment. Item1: I am in a situation I feel trapped in, item 2: I have a strong desire to escape from things in my life, item 3: I am in a relationship I cannot get out of, item 4: I often have the feeling that I would just like to run away, item 5: I feel powerless to change things, item 6: I feel trapped by my obligations, item 7: I can see no way out of my current situation, item 8: I would like to get away from other more powerful people in my life, item 9: I have a strong desire to get away and stay away from where I am now, item 10: I feel trapped by other people, item 11: I want to get away from myself IE, item 12: I feel powerless to change myself, item 13: I would like to escape from my thoughts and feelings, item 14: I feel trapped inside myself IE, item 15: I would like to get away from who I am and start again, item 16: I feel I’m in a deep hole I cannot get out of.

Furthermore, the percolation statistic was estimated to determine which items belong to more than one group in the network, as shown in **Figure 2**, where the same three communities were detected as in the previous network. The men’s network resulted in three percolated items, where both item 12 “I feel powerless to change myself” and item 16 “I feel I am in a deep hole I cannot get out of” were found to overlap between the external (A) and internal (C) entrapment clusters, while item 3 “I am in a relationship I cannot get out of” belonged simultaneously to the external (A) and external-interpersonal (B) entrapment groupings. On the other hand, in the women’s network, two percolated items were observed, where item 16 was common between the external (A) and internal (C) entrapment groups, and item 9 “I have a strong desire to get away and stay away from where I am now” corresponded to both external (A) and external-interpersonal (B) entrapment groups.

**Figure 3:**
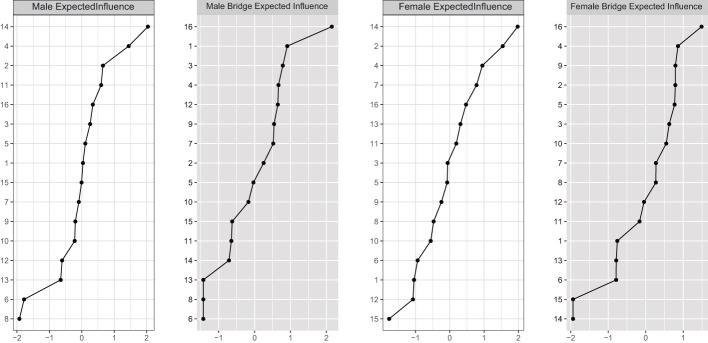
Network centrality indices.

Regarding centrality analyzes, **Figure 3** shows that the highest expected influence indexes (influential items in the whole network) were similar in men and women, represented by item 14 “I feel trapped inside myself IE” (internal trapping) and item 2 “I have a strong desire to escape from things in my life” and item 4 “: I often have the feeling that I would just like to run away” from external trapping. Regarding the highest values of bridge expected influence (items interconnected between groups), item 16 of internal entrapment “ I feel I’m in a deep hole I cannot get out of” stood out in both networks.

Comparison analyzes (NCT, Network Comparison Test) revealed that structure (M=0.11, p=0.43), strength (M=0.12, p=0.60), and centrality (M=0.11, p=0.52) were invariant, that is, there were no significant differences in estimated networks. Finally, we found at least nine significantly different associations between the two networks, as shown in **Table 2**.

**Table 2:** Edge network differences.

Var1	Var2	p-value
item 3	item 7	0.00
item 2	item 5	0.00
item 3	item 10	0.01
item 5	item 8	0.01
item 13	item 14	0.01
item 9	item 14	0.02
item 8	item 10	0.03
item 4	item 13	0.04
item 6	item 10	0.04

## Discussion

In recent studies, there has been increasing interest in the transdiagnostic significance of the concept of entrapment in connection with various psychological disorders. These include, but are not limited to, depressive disorders, anxiety disorders, posttraumatic stress disorder, and contemplation of suicide (42). Although there is previous research that has documented differences between men and women in relation to entrapment, it is important to note that this study distinguishes itself by being a multinational investigation that focuses on specifically assessing gender disparities in the concept of entrapment through network analysis.

### Dimensions of the entrapment network

In general, the comparative results of this study indicate that the structure, strength, and centrality of the entrapment item networks are invariant between males and females. However, it is important to note the presence of other relevant findings. The theoretical framework established by Gilbert and Allan (1) identifies two areas within the concept of entrapment: internal and external. However, in the current study, in which separate networks were developed for men and women, three clusters were identified. The first cluster was composed of six items reflecting the internal domain (items 11,12,13,14,15,16). On the other hand, the external domain was divided into two distinct clusters. The first cluster included items 1,2,4,5,6,7,8,9, which seem to capture more general aspects of external entrapment. In contrast, the second cluster, called interpersonal, included items 3,8,10. This last clustering can be explained by the fact that these items share the characteristic of implying a sense of entrapment related to the influence of interactions on interpersonal dynamics. For example, in item 3 (“I am in a relationship I cannot get out of”), the orientation to feel trapped in relationships that generate discomfort and do not provide the desired peace of mind or satisfaction is reflected. This includes relationships with friends, family members, partners, or other individuals where the individual feels vulnerable due to the perception that these relationships do not contribute positively to their emotional well being (43). Similarly, item 8 (“I would like to get away from other more powerful people in my life”) involves the perception of how others can have a great influence on the individual’s life, as well as the difficulty he or she may experience in ending negative relationships or selecting people who contribute to subjective well-being, leading to a sense of lack of autonomy and control. Precisely, learned helplessness, which involves feeling powerless due to the lack of control over the immediate environment, may contribute to this feeling of entrapment (44). Finally, item 10 (“I feel trapped by other people”) may be indicative of various realities that individuals may be experiencing. Although in some cases this feeling may arise in dependent relationships where both men and women face ongoing conflict, and in certain situations these relationships can escalate into repeated violence, exacerbating the feeling of emotional entrapment (45). It is important to recognize that the reasons for feeling trapped by other people are very heterogeneous. These may include, but are not limited to, the responsibility of caring for an elderly parent, having a child with high support needs, or feeling unable to choose a desired career due to family influence (46). Thus, although our analysis suggests links between the claim of feeling trapped by others and specific relationship contexts, it explicitly acknowledges the wide range of experiences and circumstances that can influence this perception.

On the other hand, the first set, which is also within the domain of external entrapment, presents items that share characteristics centered on external stimuli that increase the feeling of entrapment, but in divergence with the second set, it does not focus on the feeling of entrapment by interpersonal ties, but by the circumstances and events that occur. For example, the items focused on situational agents and obligations (item 1 “I find myself in a situation in which I feel trapped”, item 6 “I feel trapped by my obligations” and item 7 “I see no way out of my current situation”) highlight the sense of entrapment that an individual may experience when faced with external demands that are beyond his or her immediate control, which is more oriented towards work, family and personal responsibilities, and not being able to avoid these obligations can lead to increased psychological distress, worry, negative affect and maladaptive coping strategies (9). Likewise, this set considers indicators that focus on the desire to escape from the perception of entrapment (item 2 “I have a strong desire to escape from things in my life”, item 9 “I wish I could escape and stay out of where I am now”, item 4 “ I often have the feeling that I would just like to run away “), in this way, the individual wishes to escape from situations that are perceived as negative or uncomfortable, i.e. escape from the present reality (47). Finally, item 5 (“I feel powerless to change things”) reflects an individual’s hopelessness when they feel that external circumstances do not allow them to make a positive change to free themselves from entrapment, implying resignation to the situation that may lead to an increased risk of self-destructive thoughts or behaviors (47).

### Divergent results in both networks

Regarding the results of higher degree relationships between indicators from different network domains, it is found that item 4 (“I often have the feeling that I would just like to run away”) linked to external entrapment and item 13 (“I would like to escape from my thoughts and feelings”) identified in internal entrapment show an interconnection only in the women’s network. These results show the intention of wanting to escape from adverse situations, without necessarily implying an immediate action to achieve it, which seems to be accentuated due to the persistence of such situations or the expansion of the feeling of entrapment to other areas of life, including family, work, or academic (48). The intensification of this sensation is associated with the perception of restricted control, both externally and internally. In essence, individuals feel a lack of control over their circumstances, causing them to frequently consider their life events and emotional state. It is important to acknowledge that these perceptions can stem from tangible and objectively challenging circumstances, such as confronting a terminal illness or being close to individuals from whom one cannot easily distance themselves. In this context, it is important to recognize that responses and perceptions are not necessarily irrational, but rather understandable reactions to complex and often immovable circumstances. In fact, there are empirical precedents regarding women and higher scores in ruminative processes, which makes them more susceptible to perceive external vulnerabilities (49). This perception of vulnerability, in turn, complicates their management of their personal resources and undermines their efforts to take proactive action in response to adverse circumstances (50). Instead of categorizing these perceptions as irrational, it is fundamental to understand them as a component of an adaptive response to situations that are perceived as limiting or insurmountable. This highlights the need for approaches that acknowledge and validate the complexity of entrapment experiences.

### Women’s network

On the other hand, only in the women’s network was it evident that indicator 8 (“I would like to get away from other more powerful people in my life”) had three unique associations with items 5, 6 and 9, respectively. This implies that women often feel trapped by the influence of other individuals in their lives, who come to exert significant pressure or control over them, unlike men. In this context, item 8 reflects the desire to move away from these dominant influences. This indicator aligns with item 5 (“I feel powerless to change things”) which reflects that despite their desire to distance themselves from influential individuals, they may feel powerless to do so and may be due to several reasons, such as strong emotional ties to those individuals, economic dependence or fear of the repercussions of distancing themselves from such significant relationships (45). Similarly, item 8 in relation to item 6 (“I feel trapped by my obligations”) contextualizes the situation when these influential individuals are also associated with obligations and responsibilities that a woman feels she cannot shirk (such as roles traditionally assigned to women, like family care or household chores), where the feeling of entrapment is intensified (48). Finally, item 8 interconnected with item 9 (“I have a strong desire to get away and stay away from where I am now”) explores women’s desire to get away from the influential individuals in their lives and is not limited only to physical distance, but may also comprise a deep longing to escape from the totality of their current situation, the pressures, and conflict they feel.

Likewise, among the unique findings in the men’s network, it stands out that item 4 (“I often have the feeling that I would just like to run away”) interconnects with item 15 (“I would like to get away from who I am and start again”) of internal entrapment. This finding suggests that external adverse factors, such as unfavorable working conditions or pressure to meet sociocultural expectations regarding the male sex, and internal factors, such as a sense of guilt or rejection toward one’s existence, drive the feeling of entrapment and attempted escape (51). In the case of men, the pursuit of a high social status and a sustainable economy, together with expectations related to emotional detachment as a mechanism to overcome adverse situations, can generate feelings of guilt in case of not meeting such goals (52). In addition, if these expectations or standards are rigid and self-imposed, they can intensify the feeling of entrapment. As a result, to meet both personal and social demands, men may consider drastic solutions that are often more difficult to realize, such as the idea of starting over (53, 54). This promotes a more distressing feeling that exacerbates the lack of emotional support and the devaluation of emotions, both in dealing with yourself and in interactions with others (45). This lack of approach is closely related to mental health problems, as it leads to exacerbating problems such as depression and anxiety in the male population.

### Men’s network

Likewise, another identifiable interconnection in the male network of greater interest was between item 7 (“I can see no way out of my current situation”) and item 12 (“I feel powerless to change myself”). The perception that there is no apparent solution to external and uncontrollable challenges reflects how men can face difficult situations that seem to have no solution in sight. This may be related to social pressures and expectations that urge men to face problems autonomously and solve them on their own, which is often seen as a manifestation of strength (53). In turn, experiencing a sense of personal powerlessness to overcome personal difficulties or internal limitations can lead to the feeling that the imposed standards cannot be changed. This may be influenced by gender expectations that often place an emphasis on independence and autonomy, which can make it difficult to express vulnerability or seek outside help (51). Overall, this interconnectedness reflects how men may feel pressure to deal with challenges on their own, which in turn can intensify the feeling of being trapped in situations that seem insurmountable.

### Centrality metrics in both networks

Other interesting findings were observed among items that interconnected several communities (bridge expected influence) or belonged to more than one community (clique percolation) of entrapment. For example, item 16 (“I feel I’m in a deep hole I cannot get out of”) was an important link in male and female networks, strengthening both items from the external (item 1: “I am in a situation I feel trapped in”, item 2: “I have a strong desire to escape from things in my life”, item 7: “I can see no way out of my current situation”) and internal (item 11: “I want to get away from myself IE”, item 14: “I feel trapped inside myself IE”, and item 15: “I would like to get away from who I am and start again”). In both male and female participants tested, this finding suggests that many people may feel deep hopelessness, frustration, and an inability to change a problematic situation in two essential situations. For example, it is possible that this sense of entrapment is especially reinforced when they perceive that their personal attempts in the face of adversity often fail and in the idea or constant motivations to escape in any way from a highly stressful context that they are unable to control (10). Thus, the above is consistent with reviews of studies reporting that both low frustration tolerance beliefs and perceived low self-control are relevant factors predisposing to strong psychological stress (55, 56). This includes depressive, anxious, or guilt symptoms that often make it impossible to evidence discernible change toward the future for the individual, who feels that he or she cannot escape negative life events, and can increase the risk of suicidal ideation (46, 57).

### The items overlapped in both networks

In terms of the most notable differences in centrality (bridge expected influence) between the two networks, it was noted that external trapping item 1 “I am in a situation I feel trapped in” was an important interconnector in the men’s network, as it was associated with items from two communities such as internal trapping item 16 “I feel I’m in a deep hole I cannot get out of” and external-interpersonal trapping item 3 “I am in a relationship I cannot get out of”. On the other hand, external entrapment item 4 “I often have the feeling that I would just like to run away” was more central in the women’s network, which had significant associations with other indicators of internal entrapment such as item 13 “I would like to escape from my thoughts and feelings” and external-interpersonal entrapment with respect to item 3 “I am in a relationship I cannot get out of”. The centrality in both networks seems to be distinguished in that men report a sense of being generally trapped in life, in situations that are difficult to resolve. This may be in line with previous studies suggesting that affective symptoms in men are manifested more through difficulties in functioning in their general daily activities (work, relationships, vices, among others) rather than direct emotional expression toward others (58). This can bestow an overall perception of great discomfort in various areas of life, from which may arise a desire to escape or start over as an indirect way of communicating the emotional distress already immersed in the individual’s life (59). In contrast, the women’s network suggests an entrapment more characterized by an intense emotional charge that contemplates flight as a strong desire to resolve and escape from a critical situation (60). This coincides with evidence that they often face the challenges of being trapped in dysfunctional relationships that include controlling behaviors and domestic abuse, to the detriment of physical and emotional well-being. This context has been widely documented in various countries (61) and can lead to women also being in dependent relationships (62). Therefore, simultaneously, they may experience both strong emotional attachments, while manifesting a desire to escape conflicting situations that negatively affect their coping mechanisms, making it difficult for them to find a way out of their circumstances, and reinforcing a higher prevalence of suicidal ideation (63, 64).

In other results from the men’s network, it was observed that item 12 “I feel powerless to change myself”, was shared between the external (reinforced by item 7 “I can see no way out of my current situation”) and internal (associated with item 15 “I would like to get away from who I am and start again”) entrapment communities compared to the women’s network. Similarly, in this same network, item 3 “I am in a relationship I cannot get out of” was part of both the external-trapping (associations with item 1 “I am in a situation I feel trapped in” and item 4 “I often have the feeling that I would just like to run away”) and external-interpersonal (relationships with item 8 “I would like to get away from other more powerful people in my life” and item 10 “I feel trapped by other people”) communities. Regarding such central items, according to previous studies, it could be hypothesized that: 1) men may experience entrapment due to the frustration of not being able to get themselves out of a problem, which challenges the very masculine roles that emphasize emotional strength or independence (59, 65). In addition, it is likely that 2) although many men may want to get out of a relationship that is difficult to sustain, they may also be reluctant to end them. On a personal level, some of these reasons may be the loss of positive partner experiences, while on a sociocultural level, not thriving or being rejected in a romantic relationship can affect the self-esteem and social status of an individual in this group. When combined, these situations can predispose to paralyzing emotional intensity that prevents them from finding solutions and, in severe cases, are also risk factors for suicidal ideation in men (66).

Finally, in the women’s network, item 9 “I have a strong desire to get away and stay away from where I am now” was distinguished from the men’s network, as it belonged to two communities simultaneously. In particular, it had stronger connections with indicators of external-interpersonal entrapment such as item 8 “I would like to get away from other more powerful people in my life” and item 10 “I feel trapped by other people”; in addition with indicators of external entrapment such as item 2 “I have a strong desire to escape from things in my life” and item 4 “I often have the feeling that I would just like to run away”. In the women’s group, this may indicate that the desire for autonomy or to withdraw when there are overwhelming stimuli is an attempt to restore well-being. This motivation may manifest itself in response to the external demands of daily life and social relationships characterized by instability with parental figures, friends, or partners (67). Therefore, it is plausible that women aspire to a change of environment where exposures to stressful events are minimized and they can count on the necessary social support to regain their emotional balance (68).

When analyzing the concept of internal interpersonal entrapment, it is important to consider its affinity with Joiner’s theory, particularly in terms of the hopelessness associated with the immutability of unmet interpersonal needs (69). Joiner suggests that hopelessness resulting from unfilled interpersonal needs can be a significant factor in negative psychological outcomes, such as suicidal thoughts (69). Our findings provide empirical evidence supporting the notion that hopelessness in interpersonal relationships plays a significant role in psychological well-being. We observed how interpersonal entrapment affects mental health, which resonates with this theory. Therefore, this highlights the need for future research on interventions aimed at addressing these forms of interpersonal hopelessness. It underscores the importance of considering the complexity of human needs for connection and belonging in promoting mental health.

Furthermore, the current study makes a significant contribution by providing a detailed understanding of the complexities of entrapment and its gender differences through a network analysis, a method that offers an in-depth look at the interrelationships between different aspects of entrapment. The research is enriched by the inclusion of a multinational sample, which provides a broad perspective on the universality and particularities of the phenomenon in different cultural contexts, particularly in developing countries. Indeed, replication of the research in these contexts may reveal unique patterns of entrapment and gender differences that are specific to the socioeconomic and cultural realities of these regions. Addressing entrapment in developing countries could help identify resilience factors and challenges, providing a basis for designing more effective and culturally sensitive psychosocial interventions.

### Limitations

This study has certain limitations that should be considered when analyzing the results obtained. First, it is important to note that the sample used in the six countries was not probability-based. In addition, the demographic characteristics of the participants varied among the countries examined, as both the general population and university students were included, which may limit the generalizability of the results to broader populations. Furthermore, although the number of participants was not equal in each sample, the strength of this study was the inclusion of non-Western countries that tend to receive less attention in psychological research, providing a more diverse and global perspective. On the other hand, this study presented a novel methodology for identifying differences by entrapment item connections in two different samples according to sex, which offers a more detailed comparative approach that has not been previously applied. As such, the findings should be interpreted considering this new development, where mental health prevention indicators are also reported.

## Conclusion

The multinational study demonstrated through network analysis that entrapment presents three domains in the network of men and women (internal, external, external-interpersonal). The results show invariance at the structural level of both networks; however, descriptive divergences were highlighted in the interaction of the indicators and the groupings formed, and differences in centrality were also found.

## Data availability statement

The raw data supporting the conclusions of this article will be made available by the authors, without undue reservation.

## Ethics statement

The studies involving humans were approved by the Research Ethics Committee of the Universidad Peruana Unión and did not require additional ethical clearance (Registration Number: 2022-CEUPeU-0016). The study was conducted in accordance with the Declaration of Helsinki. The studies were conducted in accordance with the local legislation and institutional requirements. The participants provided their written informed consent to participate in this study.

## Author contributions

CR: Conceptualization, Data curation, Formal analysis, Validation, Writing – original draft, Writing – review & editing. DC: Conceptualization, Data curation, Writing – original draft, Writing – review & editing. GQ: Data curation, Formal analysis, Investigation, Writing – original draft, Writing – review & editing. IH: Conceptualization, Validation, Writing – review & editing. TF: Methodology, Validation, Writing – review & editing. JO: Investigation, Visualization, Writing – review & editing. RC: Conceptualization, Data curation, Validation, Writing – review & editing. VL: Conceptualization, Validation, Writing – review & editing. ML: Investigation, Validation, Writing – review & editing. YC: Validation, Writing – review & editing, Visualization. JS: Supervision, Validation, Writing – review & editing, Visualization.

## Supplementary material

Supplementary Material Table **(A)** Associations of entrapment items in males. Item1: I am in a situation where I feel trapped, item 2: I have a strong desire to escape things in my life, item 3: I am in a relationship that I cannot leave, item 4: I often have the feeling that I would just like to run away, item 5: I feel powerless to change things, item 6: I feel trapped by my obligations, item 7: I cannot see any way out of my current situation, item 8: I would like to get away from other more powerful people in my life, item 9: I have a strong desire to get away and stay away from where I am now, item 10: I feel trapped by other people, item 11: I want to get away from myself, IE, item 12: I feel powerless to change myself, item 13: I would like to escape from my thoughts and feelings, item 14: I feel trapped inside myself, IE, item 15: I would like to get away from who I am and start again, item 16: I feel like I am in a deep hole that I cannot get out of. Table **(B)** Associations of entrapment items in females. Item1: I am in a situation where I feel trapped, item 2: I have a strong desire to escape things in my life, item 3: I am in a relationship that I cannot leave, item 4: I often have the feeling that I would just like to run away, item 5: I feel powerless to change things, item 6: I feel trapped by my obligations, item 7: I cannot see any way out of my current situation, item 8: I would like to get away from other more powerful people in my life, item 9: I have a strong desire to get away and stay away from where I am now, item 10: I feel trapped by other people, item 11: I want to get away from myself, item 12: I feel powerless to change myself, item 13: I would like to escape from my thoughts and feelings, item 14: I feel trapped inside myself, IE, item 15: I would like to get away from who I am and start again, item 16: I feel like I am in a deep hole that I cannot get out of.
