# Supplementary material for: Sex differences in entrapment in a multinational sample: a network analysis perspective
Source: Front Psychiatry. 2024 May 28;15:1321207. doi: 10.3389/fpsyt.2024.1321207 (PMC11165698; doi:10.3389/fpsyt.2024.1321207)
Supplement: Supplementary Material — Table (A) Associations of entrapment items in males. Item1: I am in a situation where I feel trapped, item 2: I have a strong desire to escape things in my life, item 3: I am in a relationship that I cannot leave, item 4: I often have the feeling that I would just like to run away, item 5: I feel powerless to change things, item 6: I feel trapped by my obligations, item 7: I cannot see any way out of my current situation, item 8: I would like to get away from other more powerful people in my life, item 9: I have a strong desire to get away and stay away from where I am now, item 10: I feel trapped by other people, item 11: I want to get away from myself, IE, item 12: I feel powerless to change myself, item 13: I would like to escape from my thoughts and feelings, item 14: I feel trapped inside myself, IE, item 15: I would like to get away from who I am and start again, item 16: I feel like I am in a deep hole that I cannot get out of. Table (B) Associations of entrapment items in females. Item1: I am in a situation where I feel trapped, item 2: I have a strong desire to escape things in my life, item 3: I am in a relationship that I cannot leave, item 4: I often have the feeling that I would just like to run away, item 5: I feel powerless to change things, item 6: I feel trapped by my obligations, item 7: I cannot see any way out of my current situation, item 8: I would like to get away from other more powerful people in my life, item 9: I have a strong desire to get away and stay away from where I am now, item 10: I feel trapped by other people, item 11: I want to get away from myself, item 12: I feel powerless to change myself, item 13: I would like to escape from my thoughts and feelings, item 14: I feel trapped inside myself, IE, item 15: I would like to get away from who I am and start again, item 16: I feel like I am in a deep hole that I cannot get out of. [file Table_1.pdf]

SUPPLEMENTARY MATERIAL

Table A. Associations of entrapment items in males

VAR	I1	I2	I3	I4	I5	I6	I7	I8	I9	I10	I11	I12	I13	I14	I15	I16
I1	0															
I2	0.19	0														
I3	0.11	0	0													
I4	0.11	0.15	0.16	0												
I5	0.11	0.16	0	0	0											
I6	0.1	0	0	0.17	0.18	0										
I7	0	0	0	0.11	0.18	0.1	0									
I8	0	0	0.24	0	0	0	0	0								
I9	0	0.18	0	0.2	0	0	0.15	0	0							
I10	0	0	0.32	0	0	0	0	0.29	0.15	0						
I11	0	0.1	0	0	0	0	0	0	0	0	0					
I12	0	0	0	0	0.17	0	0.08	0	0	0	0.18	0				
I13	0	0	0	0	0	0	0	0	0	0	0.16	0.1	0			
I14	0	0	0	0	0	0	0	0	0.09	0	0.25	0.09	0.28	0		
I15	0	0	0	0.1	0	0	0	0	0	0	0.1	0.09	0.17	0.19	0	
I16	0.17	0.11	0	0	0	0	0.16	0	0	0	0.08	0	0	0.18	0.14	0

Note: Item1: I am in a situation where I feel trapped, item 2: I have a strong desire to escape things in my life, item 3: I am in a relationship that I cannot leave, item 4: I often have the feeling that I would just like to run away, item 5: I feel powerless to change things, item 6: I feel trapped by my obligations, item 7: I cannot see any way out of my current situation, item 8: I would like to get away from other more powerful people in my life, item 9: I have a strong desire to get away and stay away from where I am now, item 10: I feel trapped by other people, item 11: I want to get away from myself, IE, item 12: I feel powerless to change myself, item 13: I would like to escape from my thoughts and feelings, item 14: I feel trapped inside myself, IE, item 15: I would like to get away from who I am and start again, item 16: I feel like I am in a deep hole that I cannot get out of.

Table B. Associations of entrapment items in females

VAR	I1	I2	I3	I4	I5	I6	I7	I8	I9	I10	I11	I12	I13	I14	I15	I16
I1	0															
I2	0.19	0														
I3	0	0	0													
I4	0.18	0.11	0.16	0												
I5	0	0.08	0	0	0											
I6	0	0.09	0	0.09	0.13	0										
I7	0.09	0	0.09	0	0.21	0.17	0									
I8	0	0	0.24	0	0.08	0	0	0								
I9	0	0.13	0	0.15	0	0	0.12	0.14	0							
I10	0	0	0.2	0	0	0.11	0	0.19	0.13	0						
I11	0	0.18	0	0	0	0	0.08	0	0	0	0					
I12	0	0	0	0	0.19	0	0	0	0	0	0.14	0				
I13	0	0	0	0.11	0	0	0	0	0	0	0.15	0.13	0			
I14	0	0	0	0	0	0	0	0	0	0	0.25	0.12	0.17	0		
I15	0	0	0	0	0	0	0	0	0	0	0	0	0.17	0.15	0	
I16	0.12	0.1	0	0	0	0	0.13	0	0	0	0	0	0	0.24	0.18	0

Note: Item1: I am in a situation where I feel trapped, item 2: I have a strong desire to escape things in my life, item 3: I am in a relationship that I cannot leave, item 4: I often have the feeling that I would just like to run away, item 5: I feel powerless to change things, item 6: I feel trapped by my obligations, item 7: I cannot see any way out of my current situation, item 8: I would like to get away from other more powerful people in my life, item 9: I have a strong desire to get away and stay away from where I am now, item 10: I feel trapped by

other people, item 11: I want to get away from myself, item 12: I feel powerless to change myself, item 13: I would like to escape from my thoughts and feelings, item 14: I feel trapped inside myself, IE, item 15: I would like to get away from who I am and start again, item 16: I feel like I am in a deep hole that I cannot get out of.
